# Left Pleural Conduit: An Alternative Route for Esophageal Reconstruction When the Anterior and Posterior Mediastinal Planes Are Inaccessible

**DOI:** 10.1016/j.atssr.2025.10.014

**Published:** 2025-11-19

**Authors:** Terrance Peng, Emily A. Cameron, Sha’Shonda Revels, Paul A. Toste

**Affiliations:** Division of Thoracic Surgery, Department of Surgery, University of California, Los Angeles, Los Angeles, California

## Abstract

Restoration of gastrointestinal continuity after esophageal resection is most commonly achieved using a posterior mediastinal or retrosternal approach. In rare cases when neither plane is accessible, thoracic surgeons are faced with limited alternatives for reconstruction. We describe a case of esophagopericardial fistula after cardiac ablation that necessitated cardiac surgery and esophageal resection. With both the posterior mediastinal and retrosternal routes rendered inaccessible, we successfully constructed a gastric conduit routed through the left pleural space by using minimally invasive techniques. This report highlights the left pleural conduit as a viable alternative for esophageal reconstruction when standard approaches are not feasible.

Restoration of gastrointestinal continuity after esophageal resection is most commonly performed through a posterior mediastinal or retrosternal (anterior mediastinal) route.^1^^,^^2^ When neither technique is feasible for reconstruction, options are limited and include subcutaneous and intrathoracic approaches.^1^^,^^2^ We present a case of esophagopericardial fistula after cardiac ablation that necessitated esophageal resection and cardiac surgery. A technical description of the eventual reconstruction with a gastric conduit routed through the left pleural space is provided.

A 66-year-old man presented 4 weeks after his second ablation for atrial fibrillation with chest pain, leukocytosis, and a computed tomographic scan demonstrating mediastinal and pericardial fluid, as well as air adjacent to the left atrium. He was transferred to our institution (Ronald Reagan UCLA Medical Center, Los Angeles, CA) and taken to the operating room on an emergency basis for suspected esophagopericardial or atrioesophageal fistula. Given the uncertainty regarding atrial communication, the initial phase of the operation was performed by the cardiac surgery team through a median sternotomy. The pericardium contained cloudy fluid, and no frank fistula was identified on opening the left atrium. However, the posterior wall was thin and discolored, thus prompting placement of a bovine pericardial patch over this region. After esophagogastroduodenoscopy and percutaneous endoscopic gastrostomy tube placement, right thoracotomy was performed. A 2-cm esophagopericardial fistula was identified. The esophagus was repaired primarily before coverage with an intercostal flap. The patient’s immediate postoperative course was unremarkable beyond a small esophageal leak, which was captured by the surgical drains. A pinhole defect was noted on endoscopy and ultimately healed approximately 3 months postoperatively.

The patient did well for several months before presenting with fever, bacteremia, and a computed tomographic scan demonstrating soft tissue thickening behind the atrium with a few foci of extraluminal air. Repeat endoscopy demonstrated a recurrent small defect at the site of the previous repair. The defect was closed using an over-the-scope clip. Although his initial postprocedural recovery was again uneventful, the patient presented a few weeks later with new neurologic symptoms and imaging findings consistent with septic emboli to the brain. Given concern for ongoing esophageal communication with the atrial patch, redo right thoracotomy and subtotal esophagectomy with cervical esophagostomy were performed.

By 8 months postoperatively, the patient recovered nutritionally, with no evidence of infection on long-term suppressive antibiotic therapy. Reconstructive options were limited given the patient’s history of extensive posterior mediastinal dissection and median sternotomy. After comprehensive discussion with multiple surgeons, the decision was made to pursue reconstruction using a gastric conduit through the left pleural route. Beginning with the patient in the lateral position, the surgical robot was used to lyse adhesions in the left pleural space and dissect the esophageal stump away from the left crus at the hiatus. After repositioning the patient supine with the left side bumped, the stomach was mobilized, and hiatal dissection was completed using a laparoscopic robotic approach. The gastric conduit was then fashioned extracorporeally through a small upper midline incision. The previous neck incision was opened and extended over the manubrium in the midline. The esophagostomy was taken down, and the head of the left clavicle, the left side of the upper manubrium, and the left first costal cartilage were resected. A thoracoscope was introduced into the left side of the chest, and a plane was developed through the sternoclavicular resection bed into the left pleural space anterior to the innominate vein and medial to the internal mammary vessels. A Foley catheter was passed inferiorly from the neck, anterior to the left hilum, and through the hiatus. The conduit was then delivered from the abdomen into the neck by using the catheter and a sterile camera bag (Figure 1). In our case, the gastric conduit was passed through the native hiatus; a separate phrenotomy would also have been feasible if the reconstruction had used an alternative conduit not requiring hiatal dissection. A side-to-side stapled anastomosis was performed using a 45-mm Endo GIA stapler (Medtronic), thus closing the common channel with running polydioxanone (PDS, Ethicon) sutures. A neck drain and jejunostomy tube were placed before closure.

**Figure 1:**
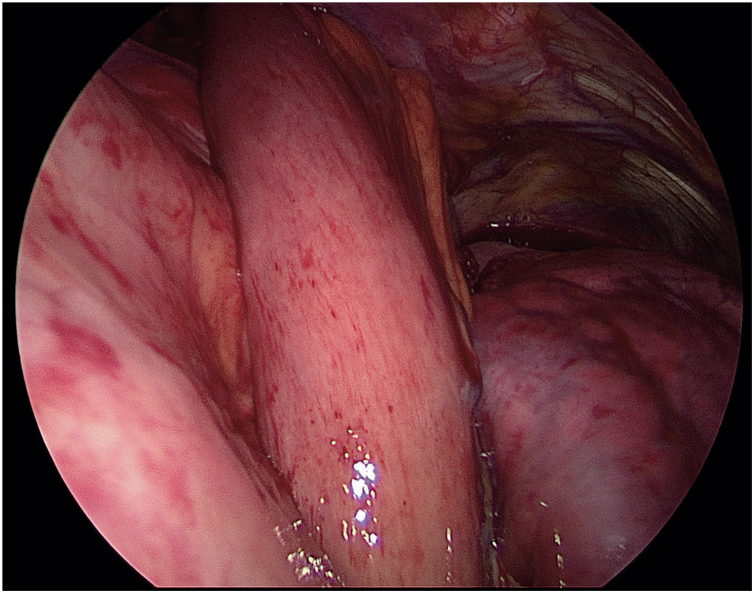
Gastric conduit in left pleural space coursing anterior to the left pulmonary hilum.

The patient recovered well postoperatively. An esophagram on postoperative day 6 demonstrated free flow of contrast medium through the conduit without evidence of leak (Figure 2). The patient was discharged on postoperative day 7. Approximately 2 weeks postoperatively, a leak developed, requiring reopening of the wound and wound vacuum-assisted closure. The wound and leak healed over a few weeks. The patient subsequently advanced to a regular diet and returned to his previous functional status.

**Figure 2:**
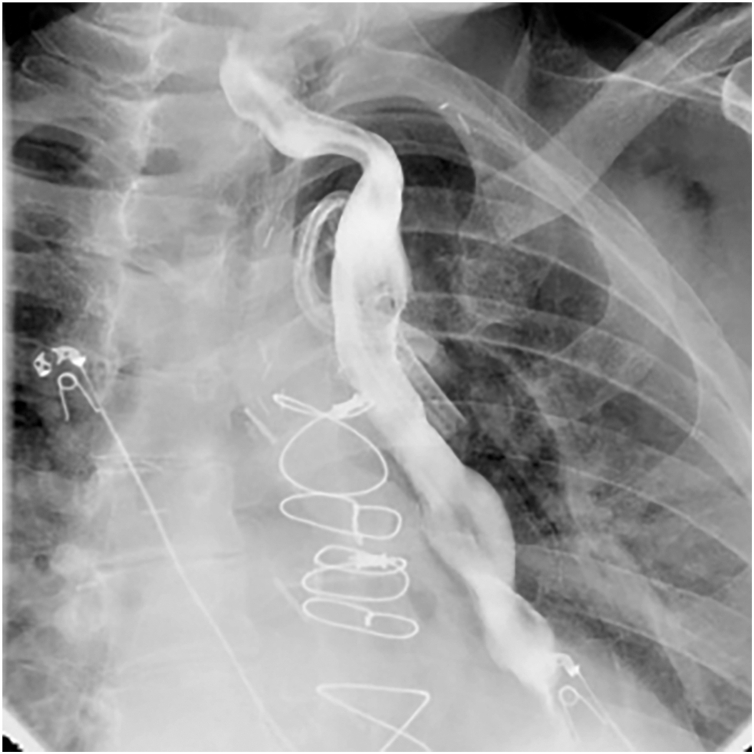
Postoperative esophagram.

## Comment

**Figure 3:**
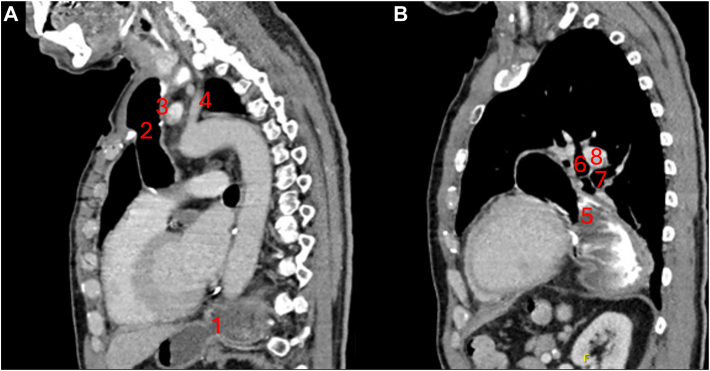
Path of gastric conduit from the hiatus through the left side of the chest and traveling anterior to the left pulmonary hilum in the midchest and anterior to the great vessels at the thoracic inlet. (A) Conduit at the hiatus and thoracic inlet: 1, conduit at hiatus; 2, anastomosis; 3, innominate vein; 4, left subclavian artery. (B) Conduit anterior to the left pulmonary hilum: 5, conduit anterior to left pulmonary hilum; 6, left upper lobe bronchus; 7, left lower lobe bronchus; 8, left pulmonary artery.

Rarely, the thoracic surgeon is faced with a patient requiring restoration of esophageal continuity in whom the posterior mediastinal and retrosternal planes are both inaccessible. In this small minority of cases, a subcutaneous colon conduit is typically chosen.^3^ This report highlights another option for reconstruction through the left pleural space (Figure 3). Use of a left pleural colon conduit was originally described by Sherman and Waterston^4^ to treat congenital tracheoesophageal fistula or atresia, but this reconstructive route is not well described in the adult population.^5^

As illustrated in this report, a well-mobilized gastric conduit can be sufficiently long to reach the neck through the left pleural space. Alternative conduits could also be used with this technique if necessary. Compared with the subcutaneous path, the left pleura offers a shorter route and greater protection of the conduit in addition to superior cosmetic results. The described approach additionally allows for a more gradual anterior-to-posterior transition from the neck to the hiatus, which may improve swallowing function and obviates the need for manual manipulation of food boluses present with subcutaneous colon conduits. This report also demonstrates that reconstruction through the left pleural space can be performed using minimally invasive techniques. From a technical standpoint, generous resection of the manubrium, clavicular head, and first rib allowed safe passage of the conduit anterior to the great vessels. Although the operation could potentially be completed with the patient in a supine position with the left side bumped, we found that starting with the patient in the lateral position for the lysis of adhesions and initial hiatal dissection provided optimal exposure. Albeit rarely needed, esophageal reconstruction with a conduit placed through the left pleural space may be a useful technique in select patients for whom standard posterior mediastinal and retrosternal routes are not feasible.
